# Success of small-dose fractionated sodium thiosulfate in the treatment of calciphylaxis in a peritoneal dialysis patient

**DOI:** 10.1186/s12882-021-02648-9

**Published:** 2022-01-03

**Authors:** Yuan Lu, Lei Shen, Ling Zhou, Deyu Xu

**Affiliations:** Department of Nephrology, The First Affiliated Hospital of Soochow University, 899 Pinghai Road, Suzhou, 215008 Jiangsu China

**Keywords:** Calciphylaxis, Sodium thiosulfate, Peritoneal dialysis, Vascular calcification

## Abstract

**Background:**

Calciphylaxis, or calcific uremic arteriolopathy (CUA), is a rare, fatal disorder of microvascular calcification and thrombosis that typically affects patients with end-stage renal disease (ESRD) receiving long-term dialysis. Fewer reports describe calciphylaxis in peritoneal dialysis patients than hemodialysis patients as per a literature review. To date, there are no clear guidelines for CUA diagnosis and treatment. While sodium thiosulfate (STS) has been increasingly used for treatment in recent years, there have also been reports of severe side effects. There is no uniform standard for its usage and dosage, especially for peritoneal dialysis patients.

**Case presentation:**

We present a case of a 40-year-old Chinese male patient with ESRD on peritoneal dialysis who developed calciphylaxis with severe painful cutaneous ulcers on the fingers and toes that were managed successfully for 6 months with comprehensive treatment composed mainly of small-dose fractionated sodium thiosulfate.

**Conclusions:**

Our experience suggests that the treatment of calciphylaxis requires timely and multi-angle intervention. Treatment with small-dose fractionated sodium thiosulfate has proven effective and tolerated in this patient.

## Background

Calciphylaxis is a severe vascular disease mainly characterized by systemic small artery calcification combined with endothelial destruction and thrombosis that can affect the skin and other organs. The injured skin site is usually accompanied by stubborn pain, which can progress to ulcers and ischemic necrosis of the surrounding tissues [1]. The pathogenesis of calciphylaxis is not yet clear, and there is no approved therapy for it. The mortality rate is high due to sepsis and internal organ failure [2], so active treatment should be initiated once diagnosed. A multidisciplinary and multifaceted approach is considered crucial in the treatment of calciphylaxis. Retrospective data reveal that sodium thiosulfate is promising as a potent calcium chelator, antioxidant, and vasodilator for the treatment of calciphylaxis, but there is no significant clinical benefit [3–5]. In addition, the optimal duration, dose, and frequency of off-label administration of STS for patients receiving peritoneal dialysis are unclear. Prospective studies are urgently needed to define the optimal treatment approach. We report here a case of a 40-year-old Chinese male ESRD patient on peritoneal dialysis, who was successfully treated by low-dose and split-course intravenous STS for calciphylaxis with skin lesions.

## Case Presentation

A 40-year-old Chinese man with a 7-year history of peritoneal dialysis of unknown etiology presented with painful necrosis of the fingers and toes for two months. He has a history of hypertension but no diabetes. Before admission, despite changing supportive dressings and taking 2 tablets of Celebrex per day, his skin ulcers continued to worsen and the pain could not be relieved. He was finally transferred to our hospital for further evaluation and treatment. He was diagnosed with secondary hyperparathyroidism 2 years before and treated with cinacalcet and lanthanum carbonate, but the effect was not good. Therefore, he underwent total parathyroidectomy and partial forearm implantation half a year prior. After the operation, he developed hypocalcemia and occasional convulsions, which were relieved by changing the peritoneal dialysis protocol and taking oral calcium gluconate. The patient's medications included metoprolo, lanthanum carbonate, compound α-ketoacid tablets, folic acid, and ferrous succinate tablets. The patient had no history of warfarin use.

**Fig. 1:**
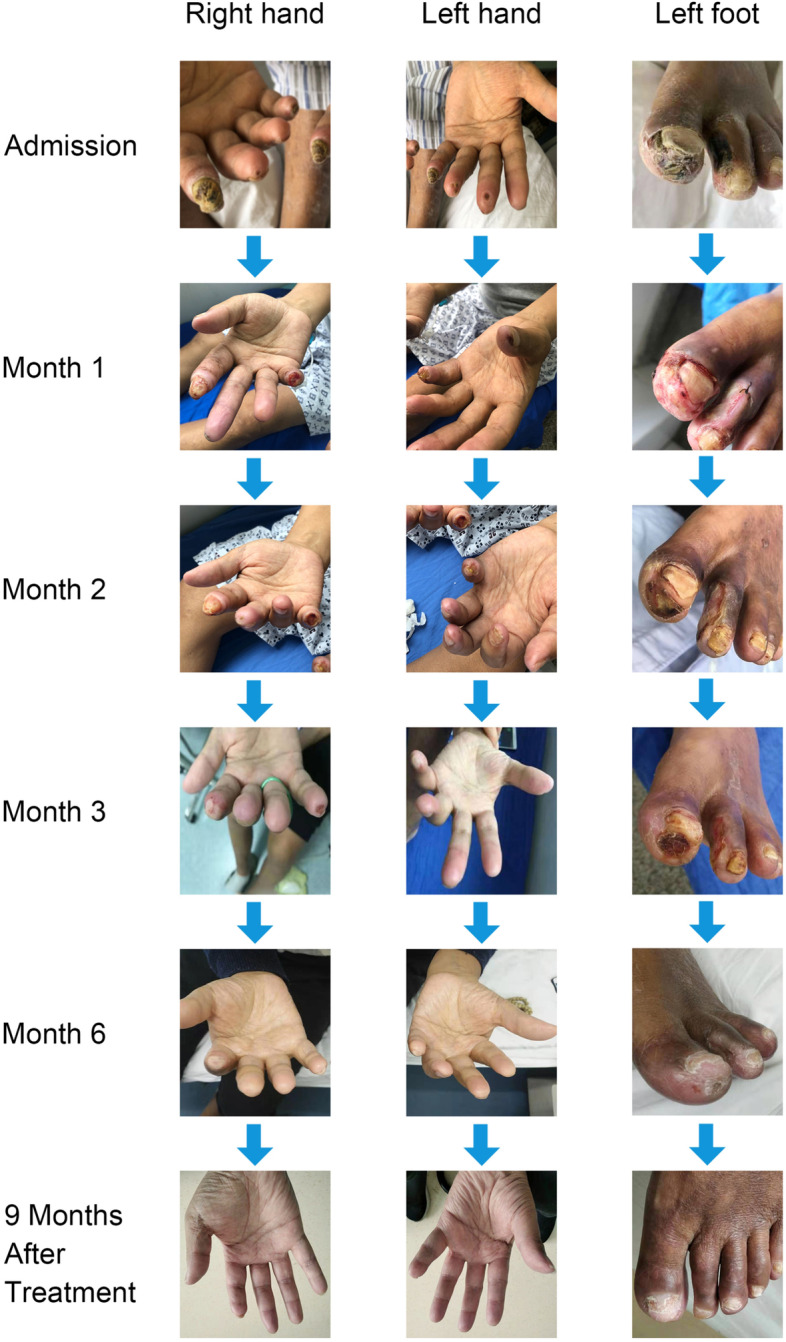
Evolution of calciphylaxis lesions over 6 months of multidisciplinary treatment and follow-up 9 months after the end of treatment

**Fig. 2:**
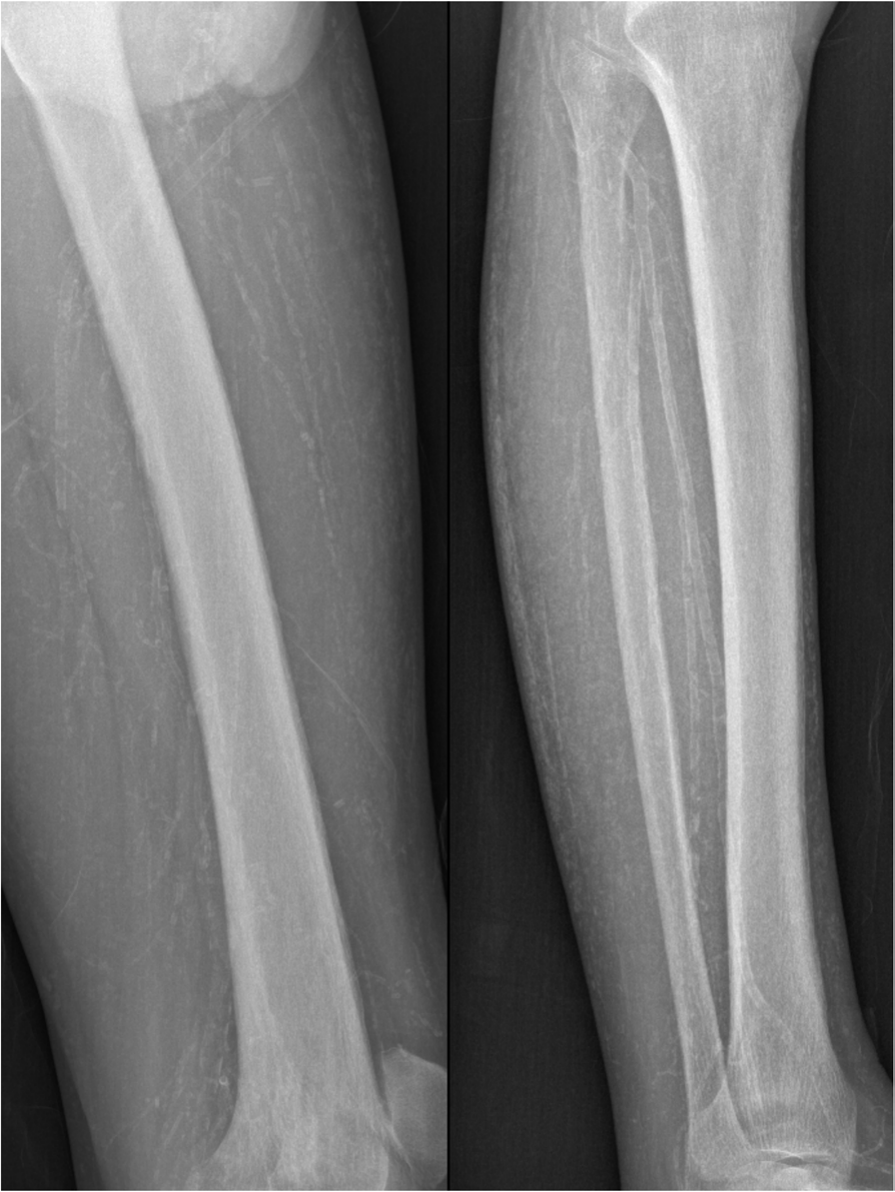
Plain radiographs of the left thigh and calf show that small-vessel calcification and a net-like pattern of calcification can be observed beyond the locus of ulcer formation

**Fig. 3:**
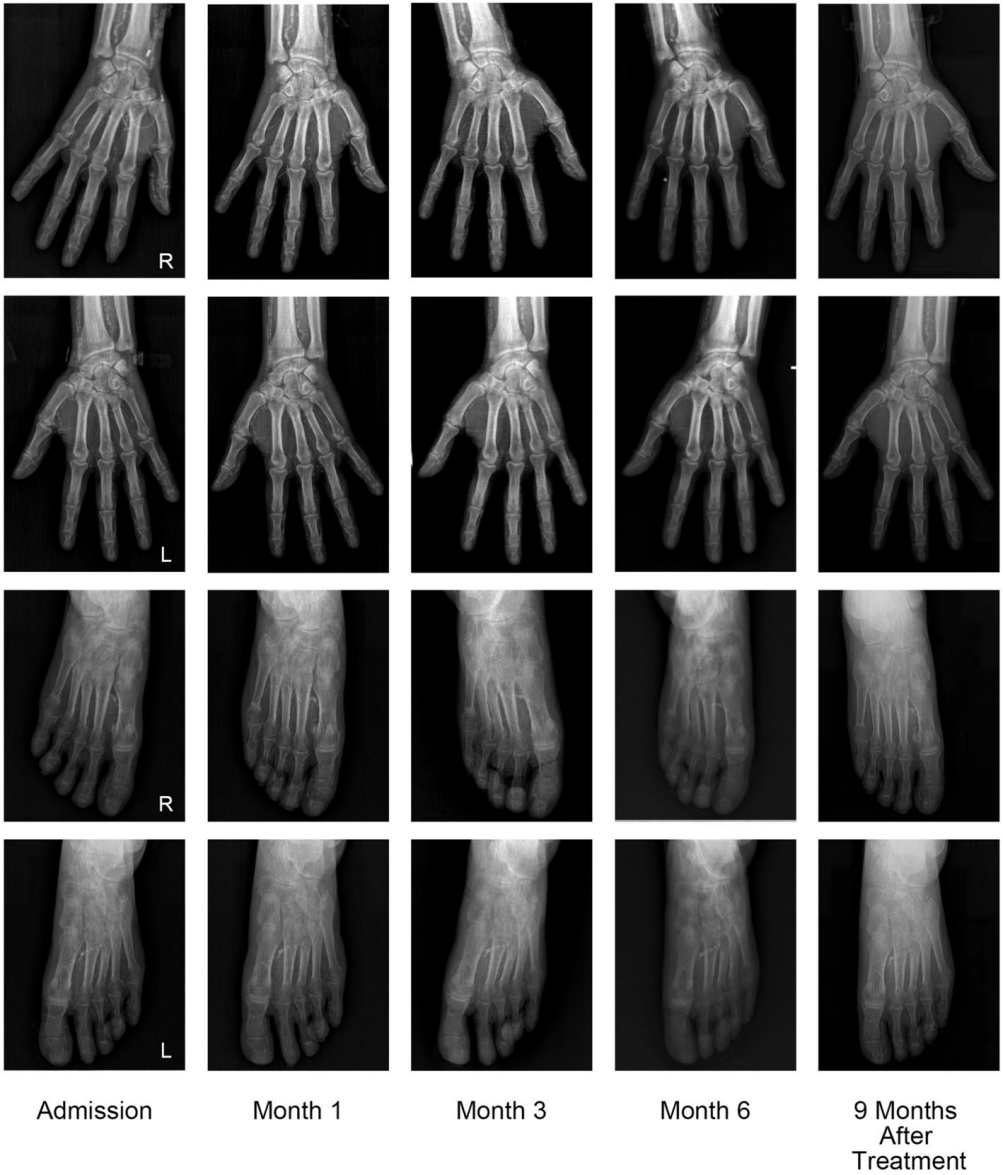
Plain radiographs of the hands and feet show severe vascular calcification and no significant reversal after treatment

On admission, his height was 173 cm, weight was 67 kg, and body mass index was 22.3 kg/m^2^. Physical examination showed necrotic ulcers of varying degrees at the tips of the fingers of both hands and toes of the left foot, covered with thick black scabs (Fig. 1). Laboratory investigations revealed the following results: white blood cell count 6200/μL, serum hemoglobin 8.8 g/dL, platelet count 184 000/μL, serum calcium 1.52 mmol/l, phosphorus 1.76 mmol/l, serum albumin 2.4 g/dL, and serum parathyroid hormone 141 pg/mL. The patient was anuric and his Kt/V urea was 1.20. The peritoneal equilibration test indicated high average transport. Plain radiographs showed extensive calcification in the upper and lower limbs, including vascular calcifications, small-vessel calcifications, and a net-like pattern of calcifications (Figs. 2 and 3). Skin biopsy during debridement from the relatively normal tissue around the ulcers in his second toe of the left foot revealed focal skin necrosis and polarization of the small artery thrombus but the absence of calcifications. We excluded other possible skin lesions based on the detailed medical history of risk factors, clinical examination, and imaging results. Calciphylaxis was considered given the various related risk factors present in our patient and the quite telling clinical presentation.

**Fig. 4:**
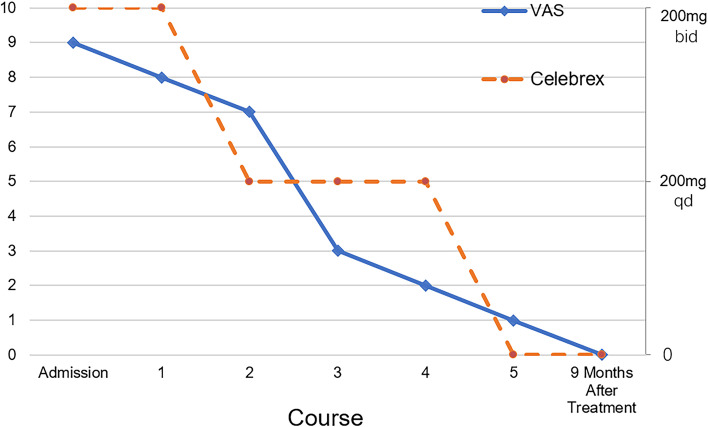
The VAS score records changes in the degree of pain during treatment and after treatment; the dose change of Celebrex as an analgesic during treatment is also reflected in the figure above

We stopped calcium supplementation and performed STS supplement therapy. The patient was treated with intravenous sodium thiosulfate at a low dose (3.2–6.4 g/d) and 5 courses, and there was a nearly 1-month intermittent period between each course. The specific prescription of the patient is shown in Table 1. The patient was managed meticulously with wound care with frequent dressing changes and periodic debridement by a burn centre and dermatology professional. According to the patient’s Kt/V urea, we adjusted the peritoneal dialysis protocol. The calcium and phosphorus metabolism was corrected by using a dialysate with a 1.75 mmol/l calcium concentration and oral lanthanum carbonate. Analgesic treatment included oral Celebrex (celecoxib capsules) twice a day, and the patient no longer needed analgesics by the fifth course. The patient reported nausea and vomiting only in the third course of treatment, which improved after medication suspension. Therefore, we adjusted the dosage of STS to 3.2 g/d in the fourth and fifth courses. After 6 months (5 courses of comprehensive treatment), the patient's skin wound had healed, and the pain was significantly relieved (Fig. 4). During therapy with STS, the clinical and biochemical parameters showed a significant decrease in white blood cells and increased hemoglobin and albumin (Table 2). In addition, no significant reversal of vascular calcification was observed on hand and foot plain films during treatment (Fig. 3). At 9 months after the end of treatment, the patient had no new skin ulcer.

**Table 1:** Prescription

Course	Duration of intravenous STS therapy (days)	Dose (g/d)	Usage	
1	28	3.2–6.4	STS (3.2 g) is diluted in 50 ml of normal saline and maintained via a venous pump for two hours	Qd
2	14	6.4		Bid
3	11	6.4		Bid
4	14	3.2		Qd
5	7	3.2		Qd

**Table 2:** Main biochemical and clinical parameters during sodium thiosulfate therapy

Parameter	Before PTX	Baseline	Course					9 months after treatment	Reference
			1	2	3	4	5		
WBC,10^9/L	7.18	6.2	6.03	5.14	5.56	5.64	5.44	4.7	3.5–9.5
RBC,10^12/L	2.41	3.50	3.84	4.23	4.46	4.44	4.51	4.75	4.3–5.8
Hb, g/L	66	88	107	110	111	107	111	120	130–175
PLT,10^9/L	316	184	181	220	172	163	163	149	125–350
NEUT%, T%	0.73	0.730	0.656	0.428	0.539	0.581	0.594	0.667	0.4–0.75
Alb, g/L	34.6	24.7	30.0	30.2	31.1	29.4	27.9	31.6	40–55
ALP, U/L	453.6	252.2	379.5	371.1	328.3	276.8	232.5	149	45–125
Ca, mmo/L	2.18	1.52	1.87	1.81	1.60	1.33	1.53	1.72	2.11–2.52
P, mmol/L	2.77	1.76	1.53	1.45	1.59	1.79	1.75	2.33	0.85–1.51
PTH, pg/ml	200	141.1	-	198.9	212.5	201.9	253.1	131	12–88

*PTX* Parathyroidectomy

## Discussion and conclusions

Calciphylaxis is a fatal systemic disease that can lead to chronic, painful, non-healing wounds and mainly occurs in patients on maintenance dialysis. Typical histopathological features are systemic arteriolar membranous calcifications, intimal fibrosis, and thrombosis[1]. Fewer reports describe calciphylaxis in peritoneal dialysis patients compared to hemodialysis patients[6]. However, some observational studies have shown that the incidence of this disease is higher in peritoneal dialysis patients than in hemodialysis patients, and the exact mechanism has not been elucidated [7, 8]. The risk factors for calciphylaxis include female sex, obesity, the number of years on dialysis, hypercalcemia, hyperphosphatemia, secondary hyperparathyroidism, drug use (such as warfarin, calcium, and iron), hypercoagulable state, vitamin K deficiency, autoimmune diseases, etc. [1, 2]. We considered the patient's long-term dialysis age, constantly abnormal calcium and phosphorus metabolism, and hyperthyroidism history as possible causes. A study based on Chinese hemodialysis patients showed that high levels of serum phosphate and alkaline phosphatase(ALP) and low levels of serum albumin at the time of diagnosis are independent risk factors for calciphylaxis [9]. Moreover, unlike previous studies in Western countries, men showed an increased risk for calciphylaxis development in this research. The patient we described also has these characteristics, which are be closely related to many factors, such as race.

Calciphylaxis can be classified as central (involving central areas within subcutaneous fatty tissue such as the abdomen or thighs) or peripheral (restricted to peripheral sites with limited fatty tissue on the digits). Central lesions are thought to be more common in dialysis patients. Our patients' lesions occurred in the extremities, and this heterogeneity of skin lesions also attracted our attention, but there is still a lack of relevant research.

There is no consensus or guidelines on the diagnostic criteria of calciphylaxis to date. A clinical diagnosis can be made for patients with high-risk factors and classic painful necrotic ulcers after excluding other diseases that cause similar skin lesion-like changes [1, 10]. Our patient presented with typical painful skin lesions on the extremities, visible vascular calcification on imaging, and obvious risk factors. After excluding atherosclerosis, diabetic ulcer, coagulation disorders, and other diseases, calciphylaxis was diagnosed.

Skin biopsy is the standard method for the confirmation of clinically suspected calciphylaxis. However, uniform histologic criteria for diagnosis have not been well established. Moreover, the histologic features of calciphylaxis can be found in unafflicted patients and therefore are not entirely specific [11]. Biopsy also has the risk of infection, delayed healing, spreading, or the formation of new ulcers [12]. We did not find histological features of typical small vessel calcification in our patient’s skin biopsies. Similar cases have been reported in the literature: calcification and other characteristic lesions may be missed because of the histological features of CUA that are punctuated in the subcutaneous tissue [13]. The pathological diagnosis of calciphylaxis usually needs multiple biopsies. Given the risk of infection and the urgent need for timely treatment in our patient, we performed only one skin biopsy during debridement. Recent studies have also confirmed that skin biopsy has a high false-negative rate, which may be related to interpretation and/or sampling issues [11, 14]. The related factors mentioned above are likely to cause a negative biopsy result in our patient. With its role in practice being debated, many scholars believe that the diagnosis of calciphylaxis requires a comprehensive evaluation of the disease in conjunction with a discussion among multidisciplinary experts and clinicopathology. Skin biopsy should be performed for suspected atypical cases (such as early nonulcerative lesions or no chronic kidney disease) [1, 13].

Non-invasive imaging is receiving increasing interest in the diagnosis of calcification. The results of a multi-centre retrospective study supported that the presence of vascular calcification on plain radiography was high sensitivity. A net-like pattern of calcification is a notable feature that may aid in diagnosis [15]. Furthermore, Charles et al. [16]reported that imaging modalities could reveal subcutaneous small vessel calcification in patients with calciphylaxis before histopathological diagnosis. Our case supports these findings and indicates that radiographic imaging can play an essential role for cases of calciphylaxis in which skin biopsy demonstrates thrombotic occlusion and ischemic necrosis but fails to reveal medial arteriolar calcification. Moreover, the plain film of our patient shows that vascular calcification extends beyond the visible skin lesions, which confirms that calciphylaxis is a systemic disease with vessel calcification.

It is currently believed that calciphylaxis treatment requires a multimodal and multidisciplinary approach, including risk factor management, pain management, wound care, medical therapy, and hyperbaric oxygen therapy. Sepsis caused by wound infection is the leading cause of death in patients with calciphylaxis, so wound care is critical. We optimized the dialysis regimen according to the patient's calcium and phosphorus levels to maintain an appropriate balance between calcium and phosphate. We also stopped oral calcium on time to reduce the risks of progression and worsening of calciphylaxis. Given the multiple severe skin ulcers on our patient’s extremities, we provided aggressive local wound care, including removing necrotic tissue, frequent dressing changes, and topical ointment to promote healing, in cooperation with dermatologists and professional wound care staff. Pain may be associated with ischemia and neuropathic pain, which is often challenging to treat. Nonsteroidal drugs are the primary drugs used for analgesia management. The patient's pain was significantly relieved in the second and third courses of comprehensive treatment, whose dependence on analgesics was also reduced. The apparent relief of pain improved patient compliance and quality of life and positively impacted the long-term prognosis, as confirmed at the 9-month follow-up.

Although there is no current Food and Drug Administration-approved therapy, sodium thiosulfate, primarily used to treat cyanide poisoning, is increasingly being used off-label to treat calciphylaxis. The therapeutic mechanism of STS action has not yet been fully elucidated. It has been shown to chelate calcium and be removed by dialysis while also having antioxidant and vasodilation properties [3, 17]. STS's common adverse effects include hypocalcemia, hypernatremia, Q-T prolongation, metabolic acidosis, headaches, bone density reduction, nausea, and vomiting [18]. The therapeutic dosage is recommended to dissolve 25 g of STS in 100 ml of normal saline given intravenously three times a week during the last 30 to 60 min of hemodialysis [19]. However, the dosing for patients receiving peritoneal dialysis is unclear. Some scholars have found that the use of classic STS doses in the Chinese population significantly increases the incidence of adverse reactions (such as nausea and vomiting), and patients usually cannot tolerate it [20]. Therefore, we decided to start treatment with multi-course, intravenous, low dose (3.2–6.4 g) sodium thiosulfate in our patient,according to Yuqiu Liu et al.[21]. This therapy was observed to be effective and tolerated in our peritoneal dialysis patient with CUA. The improvement of the patient's condition also confirmed the diagnosis despite equivocal biopsy results. STS is still an off-label drug used to treat calciphylaxis and requires the approval of relevant agencies and close monitoring of adverse reactions during use. Multi-centre randomized controlled trials are urgently needed to investigate its efficacy, safety, and survival benefit to develop optimal dosing regimens.

For peritoneal dialysis patients, a transition to hemodialysis has been recommended in some studies because hemodialysis may accelerate wound healing by better controlling mineral metabolism [22]. We did not perform this conversion because of our patients' poor vascular condition and the current treatment's effectiveness. We should monitor the patient's dialysis adequacy, and hemodialysis should be considered when necessary.

In conclusion, CUA is a rare disorder with a poor prognosis, and there is currently no guidance or consensus on its treatment. In the current case, we describe a patient on peritoneal dialysis with calciphylaxis. A comprehensive approach with off-label intravenous STS treatment at small dose and divided course resulted in the successful healing of skin lesions after 6 months and showed good safety. The research in this field is still insufficient, and medical workers need to create a standardized management protocol.

## Data Availability

All data generated or analysed for this case report are included in this published article.

## Abbreviations

CUA: Calcific uremic arteriolopathy
STS: Sodium thiosulfate
ALP: Alkaline phosphatase
